# Shc3 facilitates breast cancer drug resistance by interacting with ErbB2 to initiate ErbB2/COX2/MDR1 axis

**DOI:** 10.1002/cam4.5768

**Published:** 2023-03-07

**Authors:** Yun Liu, Fang Cao, Fantong Xia, Jie Li, Xiaobao Dong, Yan Guo, Jun Zhang, Qiang Zhao, Yuanyuan Liu

**Affiliations:** ^1^ Department of Pediatric Oncology, Tianjin Medical University Cancer Institute and Hospital, National Clinical Research Center for Cancer, Tianjin's Clinical Research Center for Cancer, Key Laboratory of Breast Cancer Prevention and Therapy, Department of Genetics, School of Basic Medical Sciences Tianjin Medical University Tianjin China; ^2^ Department of Thoracic Surgery The Second Hospital of Tianjin Medical University Tianjin China

**Keywords:** breast cancer, chemoresistance, COX2, ErbB2, MDR1, Shc3

## Abstract

Multidrug resistance (MDR) is a primary limitation of breast cancer chemotherapy. The common mechanism of MDR is various anticancer drugs can be effluxed by the cell membrane protein P‐glycoprotein (P‐gp). Here, we found that ectopic overexpression of Shc3 was detected specifically in drug‐resistant breast cancer cells, consequently reducing sensitivity to chemotherapy and promoting cell migration by mediating P‐gp expression. However, the molecular mechanism underlying the interplay between P‐gp and Shc3 in breast cancer is unknown. We reported an additional resistance mechanism involving an increase in the active form of P‐gp after Shc3 upregulation. MCF‐7/ADR and SK‐BR‐3 cells can be sensitive to doxorubicin after knockdown of Shc3. Our results indicated that the interaction between ErbB2 and EphA2 is indirect and regulated by Shc3, and also, this complex is essential for activation of the MAPK and AKT pathways. Meanwhile, Shc3 promotes ErbB2 nuclear translocation, followed by a subsequent increase of the COX2 expression through ErbB2 binding to the COX2 promoter. We further demonstrated that COX2 expression was positively correlated with P‐gp expression and the Shc3/ErbB2/COX2 axis upregulates P‐gp activity in vivo. Our results show the crucial roles of Shc3 and ErbB2 in modulating P‐gp efficacy in breast cancer cells and suggest that Shc3 inhibition may enhance the sensitivity to chemotherapeutic drugs that target oncogene addiction pathways.

## Graphical abstract

Our results show the crucial roles of Shc3 and ErbB2 in modulating P‐gp efficacy in drug‐resistant cells and suggest that Shc3 inhibition may enhance the response to chemotherapeutic drugs that target oncogene addiction pathways.

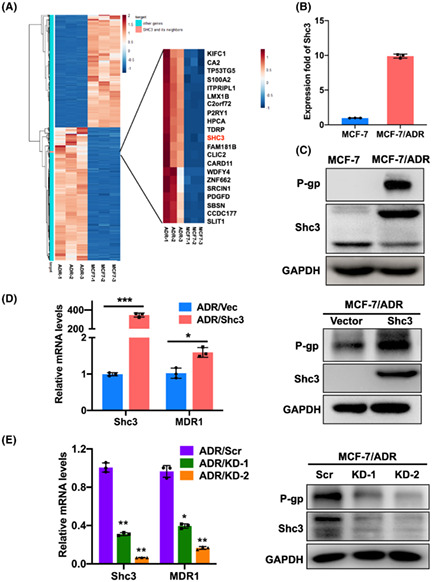

## 1 INTRODUCTION

Breast cancer is one of the most common cancers worldwide, which accounting for approximately 15% of new cancer cases, and is the second leading cause of cancer‐related mortality among females.^1^, ^2^ Among the various therapeutic strategies, chemotherapy is a major approach for advanced breast cancer patients. However, an increasing number of patients lose sensitivity to chemotherapeutics after one or two courses of treatment.^3^ Several studies have indicated that drug resistance enables cancer cells to acquire highly malignant characteristics, including epithelial‐mesenchymal transition (EMT), which is connected with invasiveness and the ability for distant metastasis.^4^ Therefore, chemoresistance of breast cancer cells leads not only to therapeutic failure but also to rapid metastasis and recurrence.^5^ Hence, identifying mechanisms of drug resistance is crucial to the development of effective cancer therapies.

Multidrug resistance (MDR) in cancer is identified by simultaneous resistance to various chemotherapeutic drugs.^6^ MDR is associated with cellular pharmacokinetic alterations, for example, augmented efflux of diverse anticancer drugs, enhanced detoxification capacity and decreased intracellular drug accumulation. Previous reports have identified several key proteins that participate in the development of MDR, including antiapoptotic proteins, ATP‐binding cassette (ABC) transporters, and DNA repair enzymes.^7^ P‐glycoprotein (P‐gp) is an important ABC transporter protein encoded by the multidrug resistance gene (MDR1) that transports a broad range of substrates, including chemotherapeutics, out of cells.^8^ However, the underlying mechanism of drug resistance involved in these regulatory processes remains insufficiently understood.

The ErbB2 proto‐oncogene is a member of the epithelial growth factor receptor (EGFR) family, which contains three other members (ErbB1, ErbB3, and ErbB4).^9^ ErbB2 is a preferred coreceptor for forming high‐affinity complexes for different ligands.^10^ Activated ErbB2 dimers can recruit various signaling molecules, leading to amplification of multiple downstream signaling cascades, such as those involving phosphatidylinositol 3 kinase (PI3K) cascades, mitogen‐activated protein kinase (MAPK) and so on.^11^ In addition, ErbB2 has been demonstrated to interact with the receptor tyrosine kinase (RTK) EPH receptor A2 (EphA2) to activate the Ras and RhoA pathways.^12^ Through the activation of these signaling pathways, ErbB2 regulates specific cellular responses, including metastasis, survival, differentiation, proliferation, and drug resistance.^13^, ^14^ In addition to its involvement in canonical signaling pathways, ErbB2 is also translocated into the nucleus, and diverse genomic targets of ErbB2, such as the cyclooxygenase 2 (COX2) and Cyclin D1 promoters, have been identified.^15^, ^16^ It is well documented that COX2 is correlated with initiation and progression of cancers. Recently, research showed that COX2 is implicated in MDR by enhancing the expression of ABC transporters, which minimize intracellular drug concentrations.^8^, ^17^

In this study, we discovered a novel role for Src homolog and collagen homolog 3 (Shc3, also called Rai, ShcC, and N‐Shc) in regulating breast cancer drug resistance. We found that enhanced Shc3 expression was detected in MDR breast cancer cell lines. Shc3 is a multifunctional adaptor protein that mediates numerous cellular processes, and its abnormal expression is associated with diverse cancers.^18^, ^19^, ^20^ We further found that Shc3 acts as a link mediating the binding between EphA2 and ErbB2 in breast cancer cells, resulting in increased activation of MAP kinase and AKT signaling pathways. Researches have shown that activation of the PI3K/Akt and MAPK pathways were attributable to MDR1 overexpression.^21^, ^22^ Furthermore, Shc3 was required for cytoplasmic‐nuclear transport of ErbB2 and promoted the binding of nuclear ErbB2 to the COX2 promoter to upregulate the expression of COX2 and P‐gp. Collectively, our findings suggested that the interaction of Shc3 with EphA2 and ErbB2 plays an important role in MDR and aggressive behavior of breast cancer.

## 2 MATERIALS AND METHODS

### 2.1 Cell lines and reagents

Human luminal‐type breast cancer cell lines MCF‐7/ADR and MCF‐7, which are, respectively, multidrug‐resistant and sensitive to doxorubicin, were kindly provided by Detroit Hospital. Cells were cultured as described previously.^23^ RPMI‐1640 medium containing 10% FBS was used for culture of the SK‐BR‐3 cell line (Gibco). All experiments were conducted with early passage cells, which were tested for mycoplasma.

### 2.2 Plasmids

The Shc3‐specific shRNA was subcloned into the pLko.1‐puro vector at the EcoRI and AgeI cloning sites (Table S1). Expression plasmids were generated by subcloning the full‐length wild‐type Shc3 cDNA fragment in the lentiviral vector pCDH‐puro‐CMV‐cDNA.

Stealth siRNAs specific to ErbB2 and COX2 were used along with a negative control siRNA (GenePharma). Tables S2 and S3 provide sequence information.

### 2.3 Quantitative real‐time reverse transcription PCR

A total RNA extraction was performed according to the previous description.^18^ PrimeScript RT Master Mix Kit was used to synthesize first‐strand cDNA (Takara). Real‐time reverse transcription PCR was fulfilled with TB Green Premix Ex Taq Kit (Takara) and measured by 7500 fast real‐time PCR (ABI). The primers were list in Table S4.

### 2.4 Transwell assays and wound healing

Transwell assay were carried out using Transwell chambers (CORNING). The upper chambers were filled with resuspended cells in 200 μL of FBS‐free DMEM, and the bottom chambers were filled with 600 μL of 10% FBS‐containing DMEM. The cells were fixed and stained after incubation for 24 h.

Cells from the experimental and control groups were plated in six‐well plates. In order to make a wound in the cell layer, a pipette tip was scraped across it after the cells were 80% confluent. Then, cell migration was measured after 0, 3, 6, 9, 12, and 24 h of incubation in medium containing 1% FBS. The wounded areas were imaged at 0 and 24 h with an IX71 inverted microscope (Olympus).

### 2.5 Western blot and coimmunoprecipitation (IP) assay

Following the procedures described previously, we performed Western blots and co‐IP assay.^18^ The detailed antibodies were described in Data S1.

### 2.6 Chromatin immunoprecipitation (Ch‐IP) assay

We used EpiQuik ChIP Kit (Epigentek) to perform the ChIP assay. According to the manufacturer's instructions, anti‐ErbB2 antibody was used to immunoprecipitate DNA‐protein complexes, followed by qRT‐PCR analysis. IgG was used as the negative control. Table S4 lists the primers specific to COX2 promoter regions.

### 2.7 Xenografted breast cancer model in mice

It was ensured that animal experiments followed the guidelines of the Animal Ethical and Welfare Committee of Tianjin Medical University Cancer Institute and Hospital. To evaluate the drug‐resistant role of Shc3, female BALB/c (4–6 weeks old) nude mice were used. In brief, a total of 20 nude mice were divided into four groups randomly (*n* = 5 mice per group), subcutaneously injected with MCF‐7/ADR‐Vector and MCF‐7/ADR‐Shc3 cells (5 × 10^6^ cells per mouse), and subjected to different treatments. After mice's tumors grew large enough to be measured, doxorubicin (4 mg/kg) was injected intravenously every other day for four consecutive times, and we monitored tumor volumes according to the formula: 0.5 × length × width^2^. Investigators monitoring tumor volumes were blinded to group allocations.

### 2.8 Statistical analysis

The statistical analysis was performed using SPSS version 23.0 software using two‐way ANOVA followed by corrections for multiple comparisons or comparisons between groups by Student's *t*‐tests with two‐tailed statistics. Statistical significance was defined as *p* < 0.05.

## 3 RESULTS

### 3.1 Increased Shc3 expression is associated with chemoresistance in breast cancer

**FIGURE 1:**
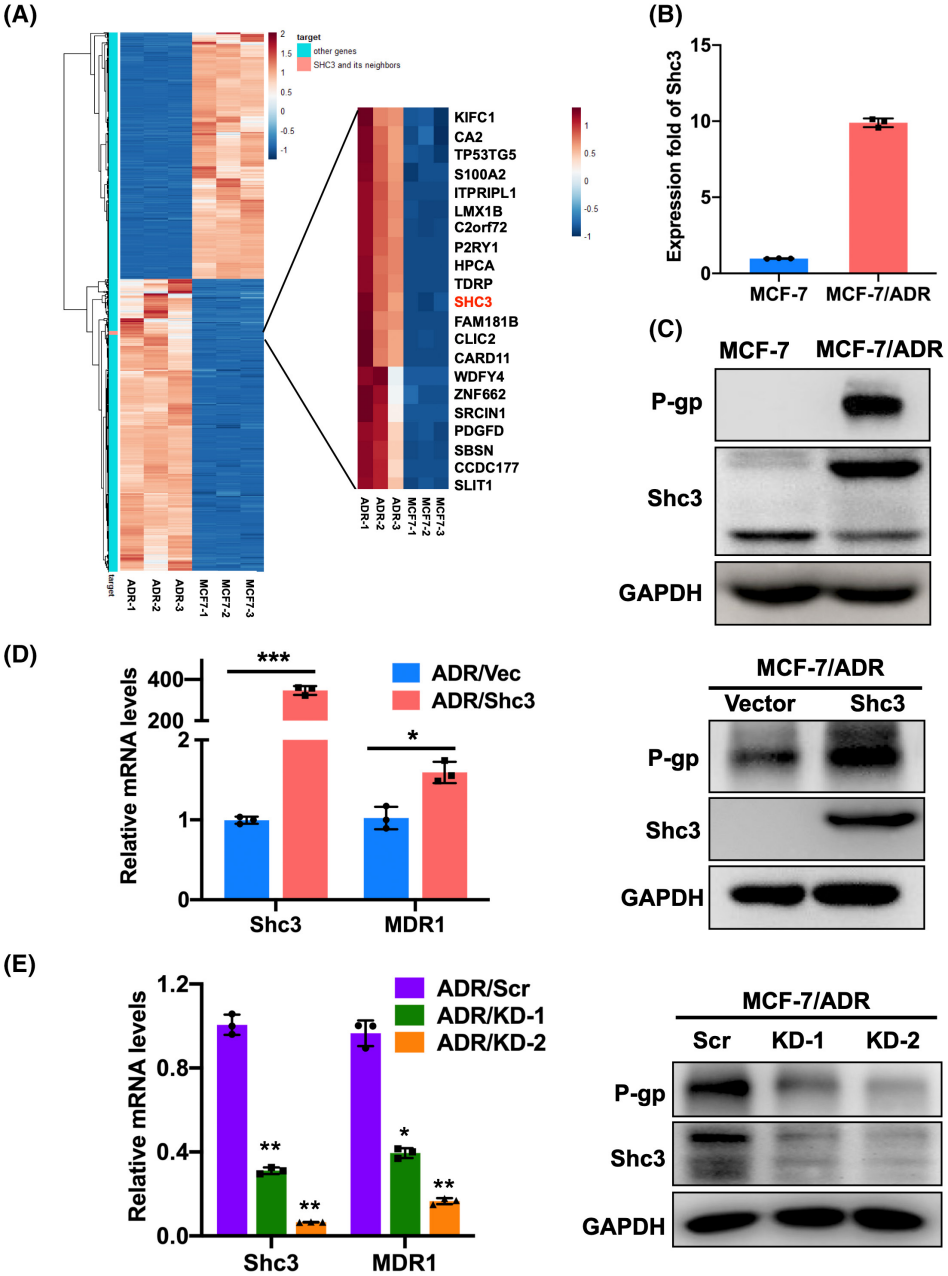
Ectopic Shc3 expression is related to chemoresistance in breast cancer. (A) Heatmap showing the differentially expressed mRNAs in MCF‐7 and MCF‐7/ADR cells. Each cell line was analyzed in triplicate. (B) The relative expression of Shc3 in MCF‐7/ADR and MCF‐7 cells was analyzed by qRT‐PCR. (C) Shc3 and P‐gp expression levels were examined by Western blot. (D) The mRNA and protein expression levels of Shc3 and MDR1 in MCF‐7/ADR stable overexpression and control cells were examined using qRT‐PCR and Western blot analysis. (E) qRT‐PCR and Western blot analysis were used to verify Shc3 and MDR1 expression in MCF‐7/ADR stable knockdown and control cells. Data are showed as mean ± standard deviation (SD) and repeated three times; NS, *p* > 0.05; *p < 0.05; **, *p* < 0.01; ***, *p* < 0.001; Students *t*‐test or ANOVA.

To identify genes that mediate breast cancer chemoresistance, we first performed differential expression analysis utilizing RNA‐seq data from human breast cancer MCF‐7 cells and a related drug‐resistant human breast cancer cell line (MCF‐7/ADR). The expression level of Shc3 in MCF‐7/ADR cells was 5.34‐fold higher than that in MCF‐7 cells (*p* < 0.05, Figure 1A). To confirm the RNA‐seq results, qRT‐PCR and Western blot analyses were performed and confirmed the marked increases in the mRNA and protein expression levels of Shc3 in MCF‐7/ADR cells compared with MCF‐7 cells (Figure 1B,C). In breast cancer, one of the most common causes of clinical resistance to doxorubicin is overexpression of ABC efflux transporters, such as P‐glycoprotein (P‐gp)/ABCB1. Patients with breast cancer expressing Shc3 have a positive correlation with P‐gp based on analysis of the R2 Genomics Analysis (http://r2.amc.nl) and Visualization Platform (Pearson's correlation coefficient 0.2, *p* < 0.0001, Figure S1A). Kaplan–Meier analysis also showed that patients with breast cancer who had high Shc3 levels had shorter overall survival (*p* = 0.0035; Figure S1B). Then, we evaluated the effect of Shc3 upregulation on MDR1 activation in three breast cancer cell lines (MCF‐7/ADR, SK‐BR‐3 and MCF‐7). As shown in Figure 1D, Figure S2A,B, MDR1 mRNA and P‐gp levels were significantly elevated in Shc3‐overexpressing cells compared with vector‐transduced cells. Consistent with this result, lower P‐gp expression was detected in Shc3‐knockdown (KD) MCF‐7/ADR and SK‐BR‐3 cells than in the corresponding control cells (Figure 1E & Figure S2A). These results reveal that elevated Shc3 expression may contribute significantly to chemoresistance in breast cancer and that Shc3 may stimulate MDR1 expression in breast cancer cells.

### 3.2 Shc3 induces chemoresistance and aggressive behavior in breast cancer cells

**FIGURE 2:**
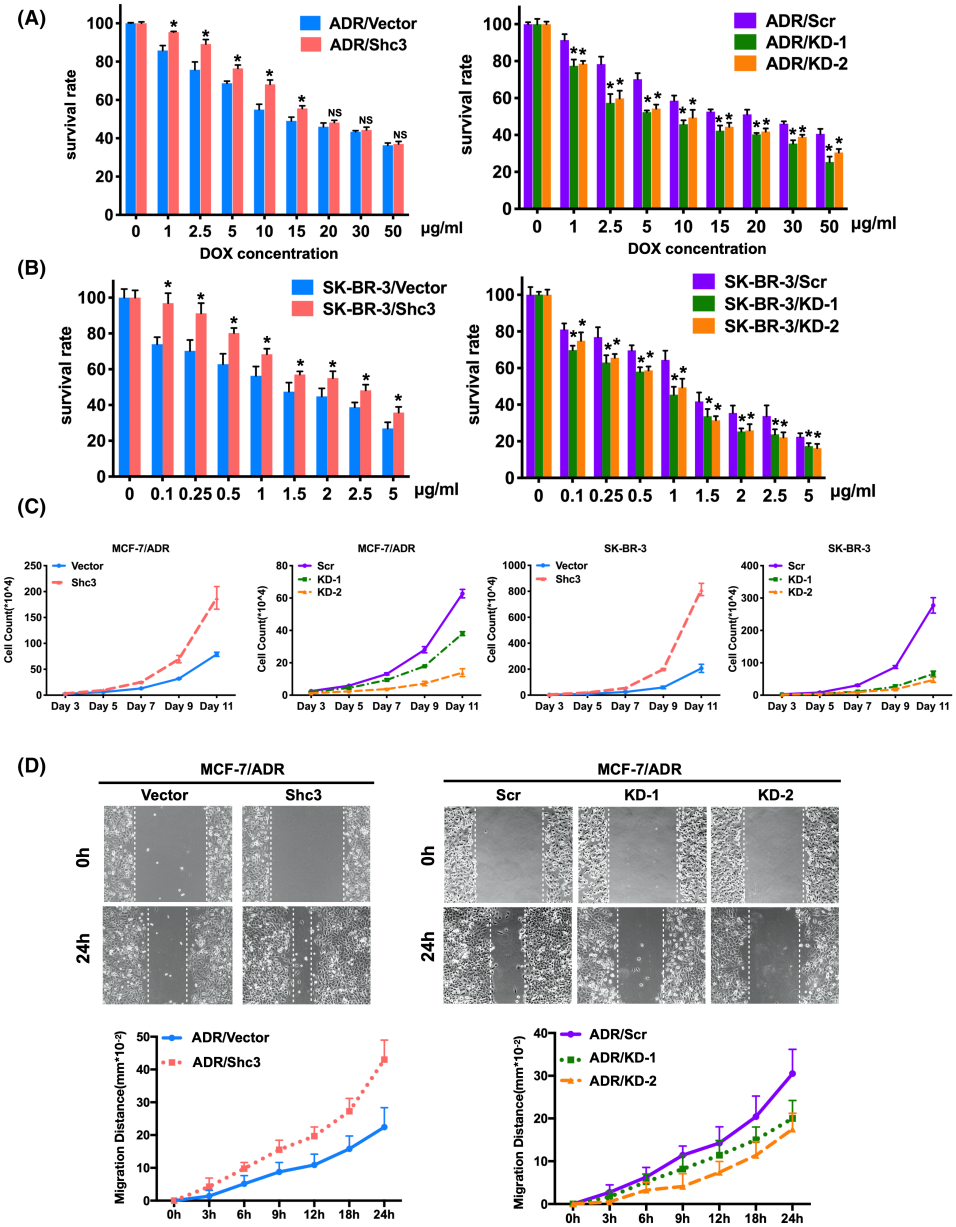
Shc3 induces chemoresistance and aggressive behavior in breast cancer cells. (A, B) The cytotoxicity of doxorubicin in cells with stable Shc3 overexpression (left) and knockdown (right) was evaluated by CCK8 assay and compared with that in the corresponding control cells. (C) Growth curve of Shc3‐overexpression and Shc3‐knockdown in MCF‐7/ADR and SK‐BR‐3 cells. (D) The wound healing of MCF‐7/ADR Shc3‐overexpressing and Shc3‐KD cells was qualitatively recorded (images was shown at 0 and 24 h, magnification: 100×). Data are shown as mean ± SD and repeated three times; NS, *p* > 0.05; *p < 0.05; Students *t‐*test or ANOVA.

The higher expression of Shc3 in MCF‐7/ADR cells indicates that Shc3 may be required for chemotherapeutic resistance. To investigate this possibility, we detected the effect of Shc3 on the sensitivity of MCF‐7/ADR, SK‐BR‐3, and MCF‐7 cells to doxorubicin by CCK8 assay. Overexpression of Shc3 decreased the sensitivity to doxorubicin in a dose‐dependent manner (Figure 2A,B left and Figure S2C). Moreover, inhibition of Shc3 notably increased the cytotoxicity of doxorubicin in drug‐resistant MCF‐7/ADR and SK‐BR‐3 cells (Figure 2A,B, right). Increased of MDR1 expression has an impact on cancer cell migration and proliferation.^24^ Hence, we explored whether Shc3 expression is involved in aggressive behavior of MCF‐7/ADR and SK‐BR‐3 cell lines. Shc3 overexpression significantly promoted but Shc3 knockdown suppressed cell proliferation (Figure 2C and Figure S2D,E). As shown in Figure 2D & Figure S2F, overexpression of Shc3 enhanced but knockdown of Shc3 markedly decreased the migration ability, as detected by wound healing assays. Taken together, these results reveal that Shc3 induces chemoresistance and aggressive behavior in breast cancer cells.

### 3.3 Shc3 interacts with EphA2 and ErbB2 to activate the MAPK and Akt pathways

**FIGURE 3:**
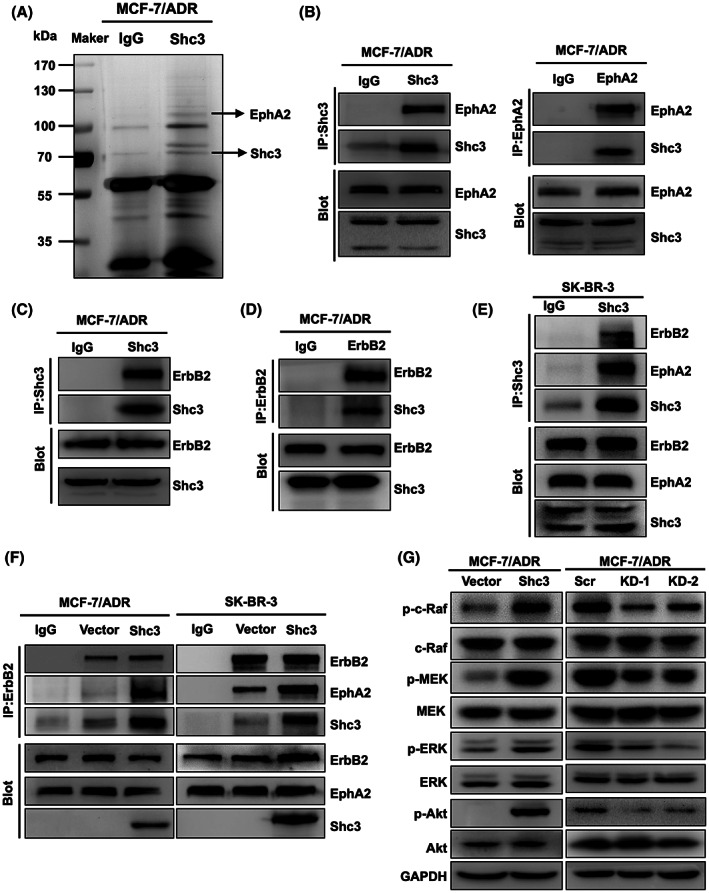
Shc3 interacts with EphA2 and ErbB2 to activate the MAPK and Akt pathways. (A) IP was performed with MCF‐7/ADR cell lysates using an anti‐Shc3 antibody. IgG was the negative control. Images of a silver‐stained SDS‐PAGE gel are shown. (B) Co‐IP assay revealing that in MCF‐7/ADR cells endogenous Shc3 interacts with endogenous EphA2. (C, D) Co‐IP assay showing that Shc3 connects with ErbB2 in MCF‐7/ADR cells. (E) Co‐IP assay revealing that Shc3 connects with ErbB2 and EphA2 in SK‐BR‐3 cells. (F) In MCF‐7/ADR and SK‐BR‐3 cells, Shc3 upregulation increased the interaction between EphA2 and ErbB2. Shc3 overexpression and vector control cells were lysed and immunoprecipitated with anti‐ErbB2 antibodies and control IgG. (G) Western blot analysis of MAPK and Akt pathway components in Shc3‐overexpressing (left panel) and Shc3‐knockdown cells (right panel).

Next, we attempt to investigate the potential mechanisms underlying Shc3‐induced chemoresistance and aggressive behavior in breast cancer. To identify the interacting protein partners of Shc3, we performed immunoprecipitation (IP) followed by mass spectrometry on MCF‐7/ADR cells and found that EphA2 was one of the top‐ranked interacting proteins (Figure 3A; Figure S3A). The interaction between Shc3 and EphA2 was further validated by a coimmunoprecipitation (Co‐IP) assay of endogenous proteins from MCF‐7/ADR cells (Figure 3B). Previous research showed that EphA2 and ErbB2 can form a complex, resulting in increased activation of the RhoA GTPase and MAPK pathways in breast cancer cells.^12^ Initially, we examined the expression of ErbB2 in MCF‐7/ADR cells. Unexpectedly, a higher level of ErbB2 expression was observed in MCF‐7/ADR cells than in MCF‐7 cells (Figure S3B). Then, we found that association between EphA2 and ErbB2 in MCF‐7/ADR cells (Figure S3C). Moreover, the interaction of Shc3 with ErbB2 in both MCF‐7/ADR and SK‐BR‐3 cells was confirmed by co‐IP (Figure 3C–E). Hence, we hypothesized that Shc3 might act as a novel adaptor between EphA2 and ErbB2 in breast cancer cells. To investigate this speculation and to further determine the specificity of the interactions between Shc3 and EphA2 and ErbB2, we overexpressed or knocked down Shc3 expression and then performed co‐IP. From the results, we concluded that both EphA2 and Shc3 can interact with ErbB2 and that overexpression of Shc3 promotes the association between EphA2 and ErbB2 (Figure 3F). In addition, Shc3 knockdown impaired EphA2‐ErbB2 coimmunoprecipitation (Figure S3D).

The activated receptor EphA2‐ErbB2 complex consequently recruits signaling molecules and activates MAPK and PI3K/Akt signaling cascades,^12^ and PI3K/Akt and MAPK pathway activation was related to drug resistance in breast cancer and its inhibitors provided a new effective drug discovery strategy.^22^, ^25^ Therefore, we hypothesized that Shc3 could modulate the function of this complex, thereby regulating MAPK and Akt pathway activation to promote drug resistance and aggressive behavior in breast cancer cells. In support of our hypothesis, Western blot analysis revealed that overexpression of Shc3 induced a marked enhancement of c‐Raf, MEK1/2, ERK1/2, and Akt phosphorylation, while downregulation of Shc3 expression inhibited these activation (Figure 3G). These observations suggest that the interactions of Shc3 with EphA2 and ErbB2 promote MAPK and Akt pathway activation.

### 3.4 Shc3 promotes nuclear localization of the ErbB2 protein and the binding of nuclear ErbB2 to the COX2 promoter

**FIGURE 4:**
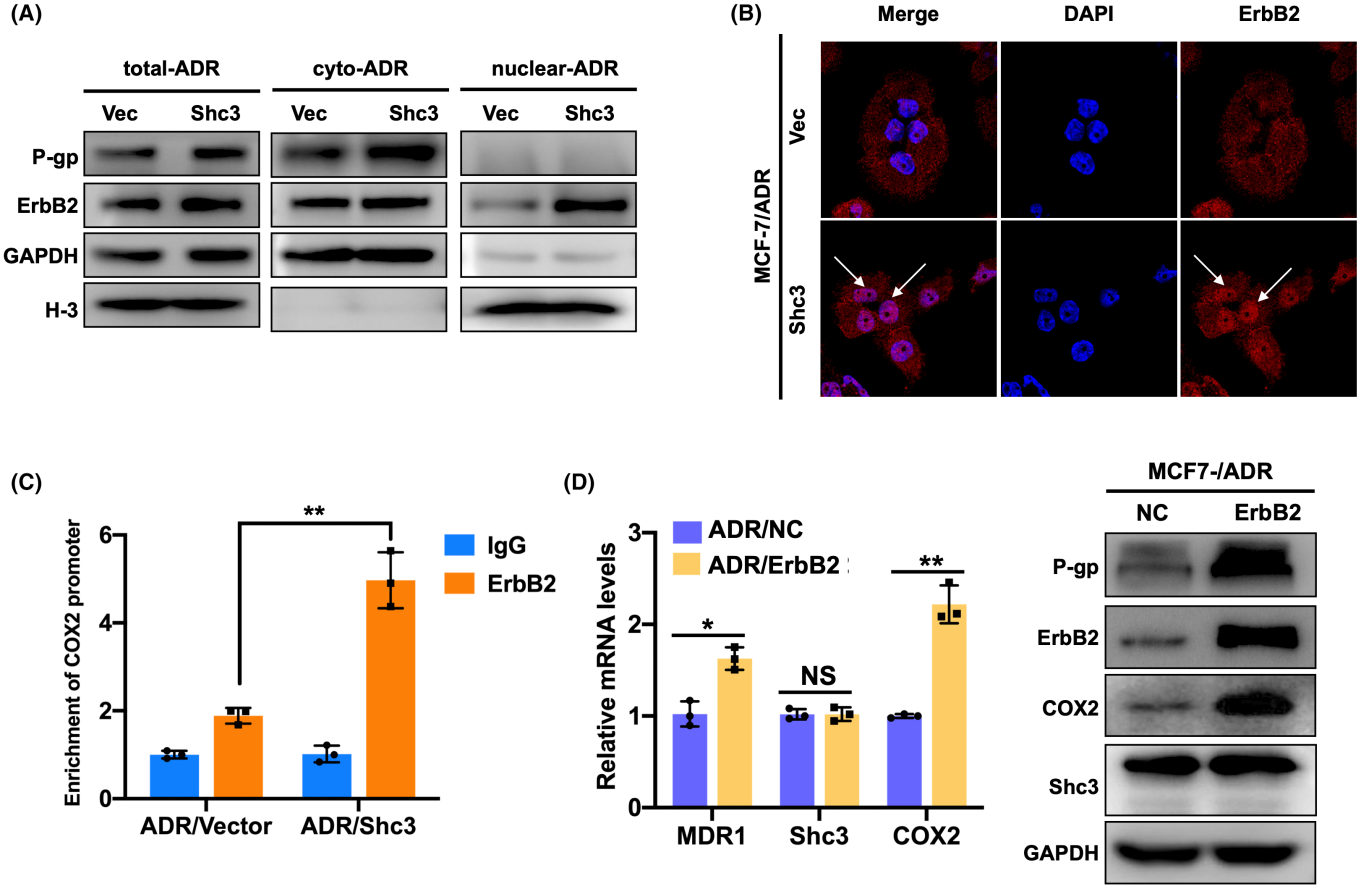
Shc3 promotes nuclear localization of ErbB2 and the binding of nuclear ErbB2 to the COX2 promoter. (A) Western blot was performed to measure the relative total, cytoplasmic and nuclear expression levels of P‐gp and ErbB2 in MCF‐7/ADR‐Vector and MCF‐7/ADR‐Shc3 cells. GAPDH was used as the cytoplasmic control; Histone‐3 was used as the nuclear control. (B) Immunofluorescence assay of ErbB2 expression in Shc3‐overexpressing and the corresponding control cells. (C) Detection of the interaction between ErbB2 and the COX2 promoter region in MCF‐7/ADR‐Vector and MCF‐7/ADR‐Shc3 cells by ChIP. (D) qRT‐PCR and Western blot were used to measure the expression of P‐gp, ErbB2, COX2, and Shc3 in MCF‐7/ADR cells transfected with the ErbB2 expression plasmid or negative control (NC) plasmid. GAPDH was used as the control. Data are presented as mean ± SD and repeated three times; NS, *p* > 0.05; *p < 0.05; **p < 0.01; Students *t*‐test.

We next investigated the effect of Shc3 expression on ErbB2 expression. Nuclear expression of ErbB2 was markedly upregulated in Shc3‐overexpressing cells compared with vector‐transduced MCF‐7/ADR and SK‐BR‐3 cells (Figure 4A & Figure S4A). Consistent with previous data, the immunofluorescence assay revealed that overexpression of Shc3 in MDR‐7/ADR cells enhanced nuclear expression of ErbB2 (Figure 4B). In breast cancer, a high expression level of COX2 is tightly associated with ErbB2 expression,^26^ and it has been discovered that nuclear ErbB2 interacts with multiple genomic targets, including the promoter of COX2 gene. Moreover, upregulation of COX2 elevates the expression of the chemoresistance‐related gene MDR1.^15^ Thus, we predicted that ErbB2 is a transcription factor that binds to the COX2 promoter region in MCF‐7/ADR cells. To test this possibility, we performed ChIP, which showed that a greater amount of the COX2 promoter region was pulled down in the anti‐ErbB2 precipitate from MCF‐7/ADR Shc3‐overexpressing cells than in that from control cells, indicating that ErbB2 binds to the COX2 promoter under regulation by Shc3 (Figure 4C). In addition, we transiently transfected an ErbB2 expression plasmid into MCF‐7/ADR cells and the results showed that up‐regulation of ErbB2 enhanced the expression of COX2 and P‐gp in MCF‐7/ADR cells (Figure 4D). Taken together, these results demonstrate that Shc3 promotes nuclear localization of the ErbB2 and is required for binding of the transcription factor ErbB2 to the COX2 promoter.

### 3.5 Shc3 regulates MDR1 via the ErbB2‐COX2 signaling pathway

**FIGURE 5:**
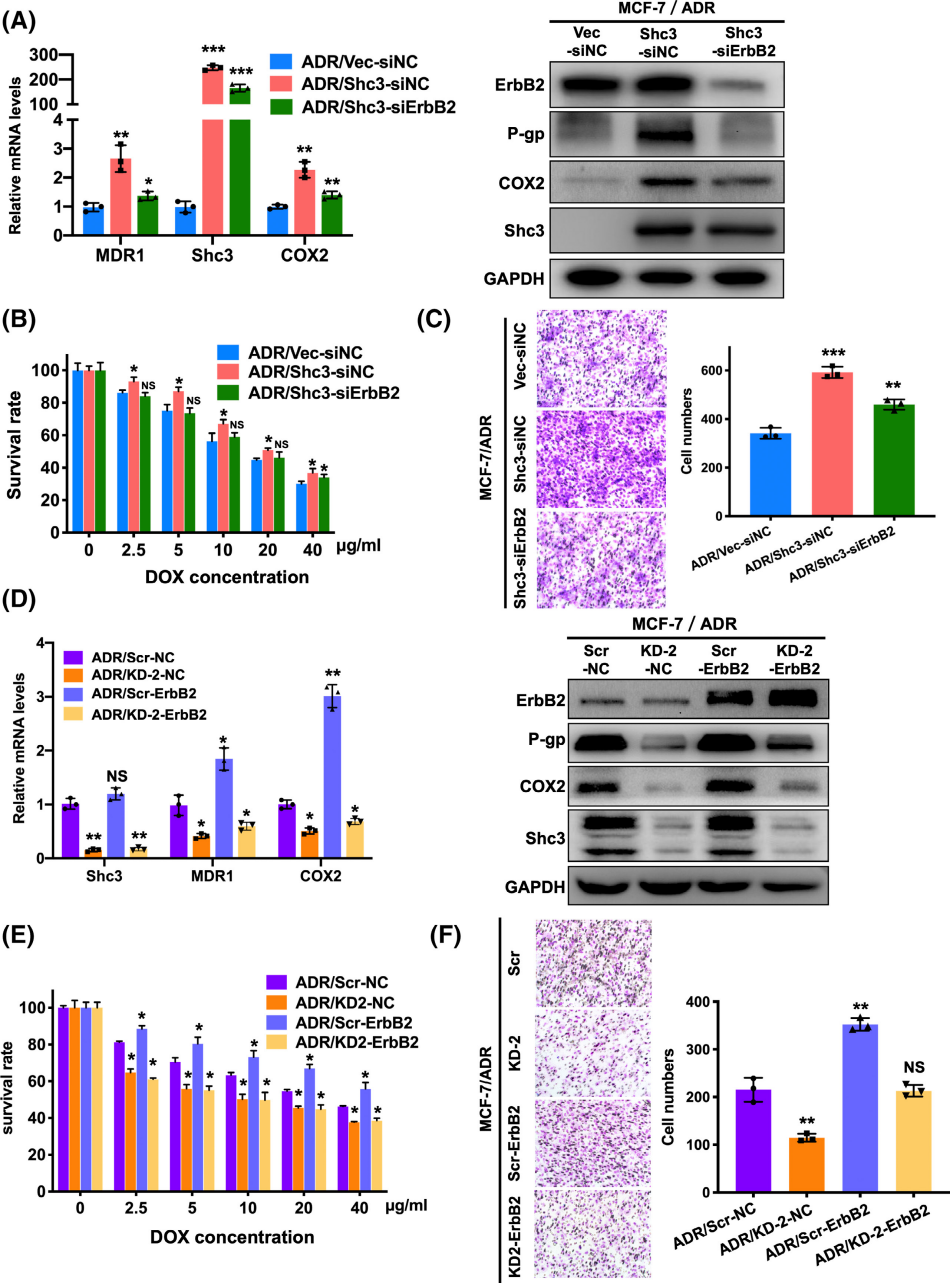
ErbB2 modulates MDR1 activation through an Shc3‐dependent pathway. (A) Downregulated expression of ErbB2 in Shc3‐overexpressing MCF‐7/ADR cells was detected by qRT‐PCR (left) and Western blot (right). MCF‐7/ADR‐Shc3 cells were treated with ErbB2 short hairpin RNA; MCF‐7/ADR‐Vector and MCF‐7/ADR‐Shc3 cells were treated with control short hairpin RNA. (B) ErbB2 inhibition in MCF‐7/ADR‐Shc3 cells restored sensitivity to doxorubicin. A CCK8 assay was used to evaluate cell viability. (C) The transwell assay showed that the migration ability of Shc3‐overexpressing cells was abrogated by ErbB2 downregulation. (D) Increased expression of ErbB2 in MCF‐7/ADR cells with stable Shc3 knockdown was observed by qRT‐PCR (left) and Western blot (right). (E) The cell viability assay showed that ErbB2 upregulation in MCF‐7/ADR control cells reduced the cytotoxicity of doxorubicin, while ErbB2 expression did not markedly affect the chemosensitivity of Shc3‐knockdown cells. (F) The transwell assay showed that overexpression of ErbB2 in MCF‐7/ADR‐Scr cells markedly enhanced the metastatic ability of these cells and partially rescued the metastatic ability in Shc3‐knockdown cells. Data are presented as mean ± SD and repeated three times; NS, *p* > 0.05; *p < 0.05; **p < 0.01; ***p < 0.001; Students *t*‐test or ANOVA.

Our previous data showed that nuclear localization of ErbB2 promotes COX2 expression by binding to its promoter in MCF‐7/ADR cells. Researches have reported that P‐gp and COX2 expression are strongly associated in many tumors and that COX2 modulates the activity and expression of P‐gp.^8^, ^27^ We hypothesized that Shc3 regulates the expression of MDR1 and then promotes drug resistance via the ErbB2‐COX2 axis. To determine whether nuclear localization of ErbB2 is necessary for the regulation of MDR1 by Shc3, we transfected Shc3‐overexpressing MCF‐7/ADR cells with siErbB2. As shown in Figure 5A, downregulation of ErbB2 inhibited P‐gp and COX2 expression in MCF‐7/ADR‐Shc3 cells. Moreover, the sensitivity of cells to doxorubicin was increased in response to ErbB2 downregulation (Figure 5B). The results of the rescued chemotaxis assays showed that ErbB2 downregulation markedly suppressed the Shc3 overexpression‐induced increase in the migration ability (Figure 5C).

These findings prompted us to further confirm whether ErbB2 modulates MDR1 activation through an Shc3‐dependent pathway. To this end, we upregulated ErbB2 expression in MCF‐7/ADR‐Scr and MCF‐7/ADR‐KD cells. The expression of both MDR1 and COX2 was effectively increased in ErbB2‐overexpressing MCF‐7/ADR‐Scr cells. However, neither MDR1 nor COX2 expression was rescued in ErbB2‐overexpressing MCF‐7/ADR‐KD cells (Figure 5D). Consistent with these results, up‐regulation of ErbB2 markedly decreased the sensitivity of MCF‐7/ADR‐Scr cells to doxorubicin in a dose‐dependent manner. Interestingly, ErbB2 upregulation had little impact on the cytotoxicity of doxorubicin in Shc3‐knockdown cells (Figure 5E). On the other hand, overexpression of ErbB2 enhanced the migration capability of MCF‐7/ADR‐Scr and MCF‐7/ADR‐KD cells compared with control cells (Figure 5F). These data imply that ErbB2 mediates MDR1 expression and drug resistance via an Shc3‐dependent pathway.

**FIGURE 6:**
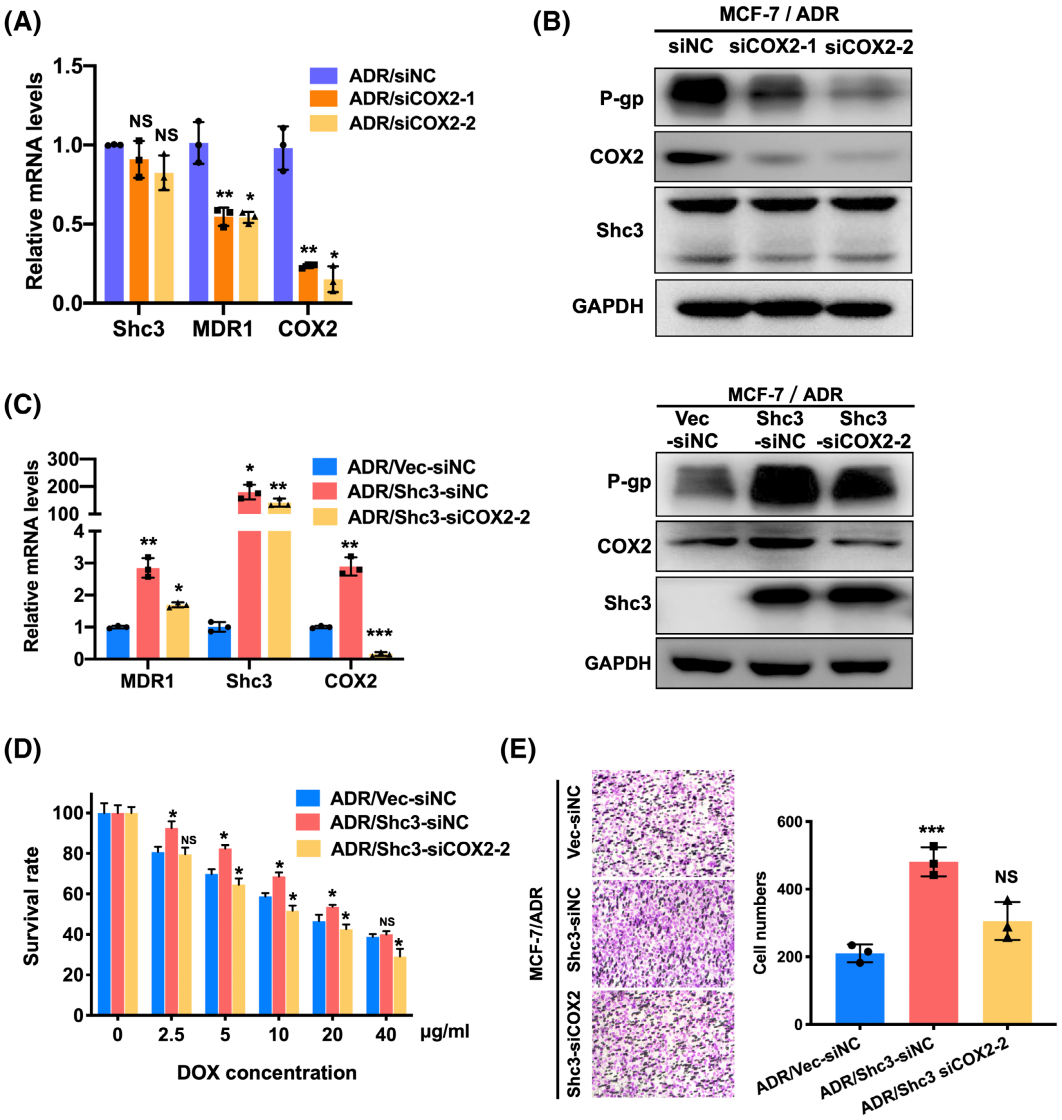
Shc3 regulates MDR1 via the ErbB2‐COX2 signaling pathway. (A) The expression of COX2 was knocked down using two siRNAs in MCF‐7/ADR cells. The mRNA expression levels of COX2, MDR1, and Shc3 were measured by qRT‐PCR. (B) Western blotting was carried out to measure the relative expression levels of P‐gp, COX2, and Shc3 in MCF‐7/ADR cells with COX2 downregulation. (C) Downregulated expression of COX2 in MCF‐7/ADR‐Shc3 cells was observed by qRT‐PCR and Western blot. (D) The cell viability assay showed that downregulation of COX2 in MCF‐7/ADR‐Shc3 cells restored doxorubicin sensitivity in a dose‐dependent manner. (E) The migration ability of Shc3‐overexpressing cells was reduced by COX2 downregulation. Data are presented as mean ± SD and repeated three times; NS, *p* > 0.05; *p < 0.05; **p < 0.01; ***p < 0.001; Students *t*‐test or ANOVA.

We next examined whether COX2 is essential for the Shc3‐dependent MDR1 activation pathway. As shown in Figure 6A,B, we transfected MCF‐7/ADR cells with siCOX2, and downregulation of COX2 markedly impaired P‐gp expression. Moreover, knockdown of COX2 inhibited MDR1 expression (Figure 6C), and the cytotoxicity of doxorubicin decreased in MCF‐7/ADR‐Shc3 cells in response to COX2 downregulation (Figure 6D). Transwell assays showed that COX2 downregulation markedly reduced the metastatic ability of MCF‐7/ADR‐Shc3 cells compared with MCF‐7/ADR‐Shc3 control cells (Figure 6E). Taken together, these results reveal that MDR1 is regulated by Shc3 through the ErbB2‐COX2 axis.

### 3.6 Shc3 promotes the drug resistance of breast cancer in vivo

**FIGURE 7:**
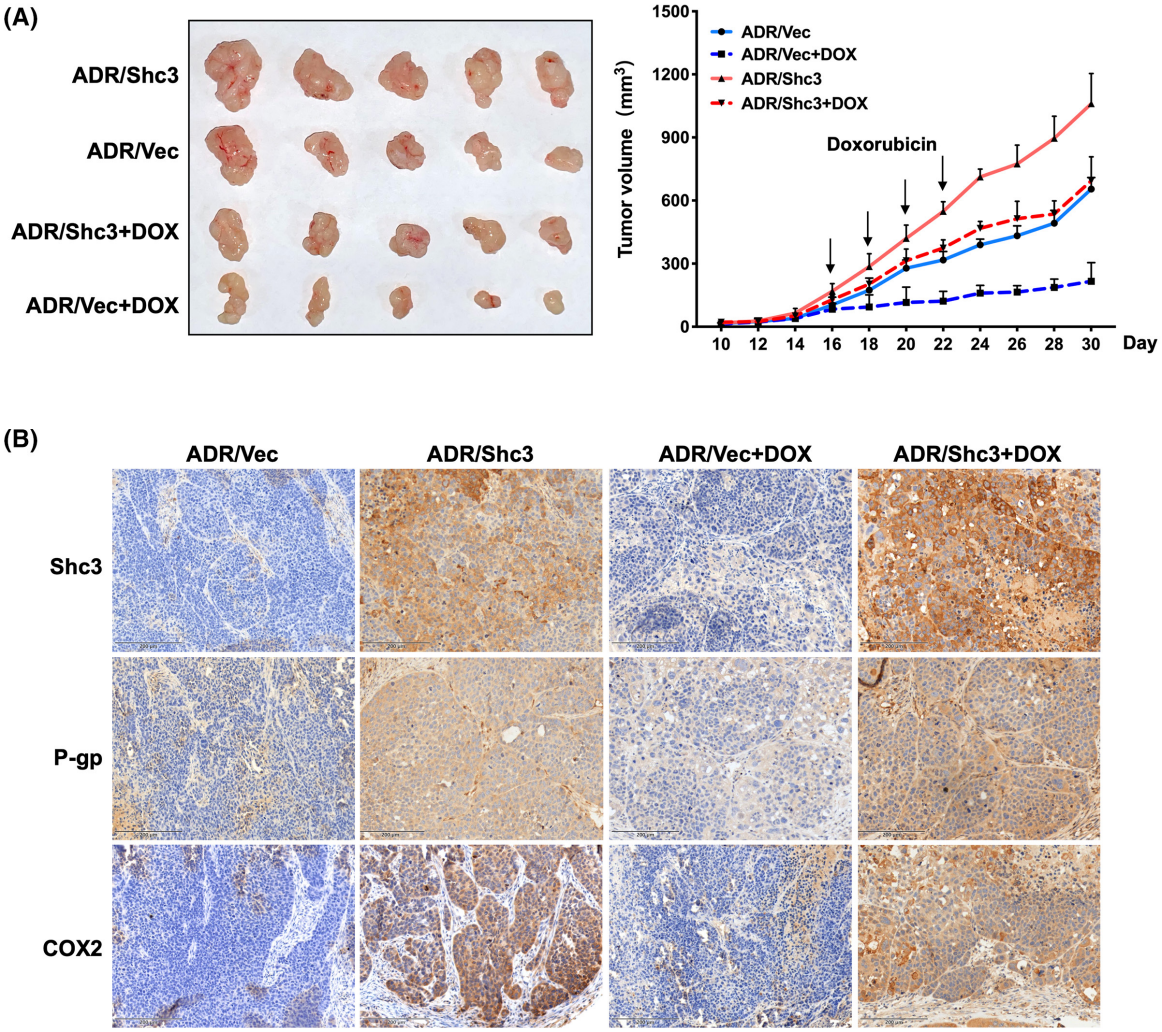
Shc3 is critical for drug resistance in vivo. (A) Photograph and tumor growth curve of different groups treated with control (normal saline) or doxorubicin in nude mice. (B) The expression levels of P‐gp, Shc3, and COX2 in tumor sections after various treatments were evaluated by IHC. Data are presented as mean ± SD, *n* = 5.

To further investigate the drug resistance potential of Shc3 in MCF‐7/ADR cells in vivo, we established a xenograft model of multidrug‐resistant breast cancer by subcutaneous inoculation of mice with MCF‐7/ADR‐Vector or MCF‐7/ADR‐Shc3 cells. Two weeks later, mice were treated with doxorubicin or normal saline (negative control) via intravenous injection for four consecutive treatments. Tumor volumes were recorded as shown in Figure 7A. Shc3 overexpression significantly promoted drug resistance. After doxorubicin treatment, Shc3 overexpression group tumor volume reduced 34.50% compared with doxorubicin untreatment MCF‐7/ADR‐Shc3 group; control group tumor volume reduced 66.95% compared with doxorubicin untreatment MCF‐7/ADR‐Vector group.

IHC analysis of xenograft tumors revealed that Shc3 overexpression induced drug resistance by upregulating P‐gp, as evidenced by the increase in P‐gp staining. Correspondingly, COX2 expression was increased in the tissues of tumors formed from MCF‐7/ADR‐Shc3 cells, especially in the doxorubicin treatment group (Figure 7B). According to these findings, Shc3 expression in breast cancer cells can impair chemosensitivity in vivo.

## 4 DISCUSSION

In many patients with breast cancer, treatment is eventually restricted by the development of acquired resistance, but the underlying mechanism has not been well explained. P‐gp has been reported to be a major protein mediating cancer cell resistance to chemotherapeutic agents. This study found that multidrug‐resistant breast cancer cells exhibit pronounced upregulation of Shc3 expression and that Shc3 regulates the expression of the MDR1 gene, a crucial factor in drug resistance. Mechanically, Shc3 acts as a novel binding protein of EphA2 and ErbB2, and Shc3 also contributes to drug resistance by facilitating nuclear translocation of ErbB2.

The SHC3 gene encodes two protein isoforms, one of 52 kDa and one of 64 kDa. Shc3 has conserved domains with a unique phosphotyrosine‐binding domain (PTB), a central region (CH1), and carboxy‐terminal SH2 modular structure, and p64Shc3 contains an additional N‐terminal CH2 domain.^28^ The PTB and SH2 domains are phosphotyrosine‐recognition modules that recognize various phosphotyrosine‐containing peptides, such as those in RTKs, EGFR, and RET.^19^ Previous research showed that formation of the EphA2‐ErbB2 complex can be promoted by ligand binding and that the phosphotyrosine‐binding domains are the PTB and SH2 domains. Hence, we suggest that Shc3 acts as a link mediating the binding between EphA2 and ErbB2. ErbB2 has been shown to be involved in the progression and development of many different tumors, especially breast cancer.^29^ Activated ErbB2 dimerization receptor complexes recruit signaling molecules, leading to activation of the MAPK, STAT, PI3K‐Akt, JNK, p38MAPK, PKC, and PLC signaling cascades.^30^, ^31^ Additionally, activation of the EphA2 receptor has been found to stimulate Ras–Raf–Erk signaling^32^ and the Akt pathway.^33^ Studies have shown that both PI3K/Akt and MAPK pathway can enhance drug resistance.^22^, ^25^ In this study, we demonstrated that Shc3 acts as a novel binding protein of EphA2 and ErbB2 linking that activated Akt and MAPK pathways to mediate drug resistance and aggressive behavior in breast cancer.

In addition to their participation in canonical signaling pathways, several studies have demonstrated that RTKs, including the ErbB family, are also located in the nucleus.^15^ Meanwhile, the nuclear RTKs are often related to high tumor grade and poor patient survival in many kinds of cancer.^34^, ^35^ A growing body of evidence indicates that ErbB2, as well as other ErbB family members and their ligands, exist and function in breast cancer.^36^ Here, we demonstrated that overexpression of Shc3 induces nuclear translocation of ErbB2. We also examined the significance of the Shc3‐ErbB2 interaction in ErbB2 transactivation and found that ErbB2 translocate to the nucleus in an Shc3‐dependent manner. The aberrant signaling of ErbB2, which is an example of resistance mechanism, leads to the activation of bypass pathway.^37^ But the relationship between ErbB2 and drug resistance mechanisms has not been clearly elucidated, our findings point to one such mechanism. Nuclear ErbB2 targets several gene promoters, including the COX2 promoter, to regulate gene expression. COX2 is a well‐known protein that mediates P‐gp expression, and its expression can dramatically upregulate P‐gp expression and minimize the cytotoxicity of doxorubicin.^38^, ^39^ Here, we verified the specific COX2 promoter as nuclear ErbB2 target in MCF‐7/ADR cells by ChIP assay. Shc3 is required for ErbB2 recruitment to the COX2 promoter, leading to MDR1 expression. Inhibition of ErbB2 or COX2 in Shc3‐overexpressing cells promoted chemosensitivity and impaired metastasis. Furthermore, induction of ErbB2 did not completely restore the MDR and metastasis properties attenuated by Shc3 inhibition, suggesting that the ErbB2‐COX2‐MDR1 axis may be dependent on Shc3 expression. These findings emphasize that Shc3 overexpression enhances ErbB2 translocation and activates the ErbB2‐COX2‐MDR1 axis, which contributes to the initiation and progression of MDR and aggressive behavior in breast cancer cells. Currently, we have not elucidated how Shc3 facilitates ErbB2 nuclear translocation, which will be investigated further in future studies.

## 5 CONCLUSION

In summary, we provide data indicating a pivotal role of Shc3 in the acquisition of chemoresistance and migration ability in breast cancer cells. Shc3 acts as a novel binding protein of EphA2 and ErbB2, and the resulting complex facilitates MAPK and Akt pathway activation. Shc3 is also required for the drug resistance breast cancer cells by mediating nuclear translocation of ErbB2, thereby facilitating P‐gp expression by promoting ErbB2‐COX2‐MDR1 axis activity. In summary, our study provides new insights into the mechanism underlying the increased activity of P‐gp caused by the upregulation of Shc3.

## AUTHOR CONTRIBUTIONS

**Yun Liu:** Data curation (lead); formal analysis (lead); funding acquisition (equal); investigation (equal); methodology (equal); writing – original draft (lead); writing – review and editing (equal). **Fang Cao:** Data curation (equal); formal analysis (equal); methodology (equal); validation (equal). **Fantong Xia:** Data curation (equal); formal analysis (equal); validation (equal). **Jie Li:** Resources (equal); supervision (equal). **Xiaobao Dong:** Methodology (equal); resources (equal); software (lead); validation (equal). **Yan Guo:** Data curation (equal); resources (equal); validation (equal). **Jun Zhang:** Data curation (equal); resources (equal); validation (equal). **Qiang Zhao:** Conceptualization (equal); funding acquisition (lead); project administration (equal); resources (equal); supervision (equal); writing – review and editing (equal). **Yuanyuan Liu:** Conceptualization (equal); funding acquisition (equal); project administration (equal); resources (equal); supervision (equal); writing – original draft (equal); writing – review and editing (lead).

## FUNDING INFORMATION

Support for this research was provided by the National Natural Science Foundation of China (82002513, 82073383), the National Key R&D Program of China (2018YFC1313000, 2018YFC1313001), and the Tianjin Key Medical Discipline (Specialty) Construction Project (TJYXZDXK‐009A).

## CONFLICT OF INTEREST STATEMENT

The authors declare that there are no known competing financial interests or personal relationships that could have influenced the work reported in this article.

## ETHICS APPROVAL AND CONSENT TO PARTICIPATE

Animals used in this study were kept in accordance with guidelines and procedures set out by the Institutional Animal Care and Use Committee of Tianjin Medical University Cancer Institute and Hospital. Animal experiments at Tianjin Medical University's Cancer Institute and Hospital were approved by Stamp of the Animal Ethical and Welfare Committee (LWFB‐AE‐2022002).

## CONSENT FOR PUBLICATION

No applicable.

## Supporting information


Figure S1.
Figure S2.
Figure S3.
Figure S4.

Table S1.
Table S2.
Table S3.
Table S4.

Data S1.

## Data Availability

For reasonable requests, the corresponding author can provide datasets generated and/or analyzed during this study.
