# Supplementary material for: Shc3 facilitates breast cancer drug resistance by interacting with ErbB2 to initiate ErbB2/COX2/MDR1 axis
Source: Cancer Med. 2023 Mar 7;12(9):10768–80. doi: 10.1002/cam4.5768 (PMC10225176; doi:10.1002/cam4.5768)
Supplement: Supplementary file 1 — Figure S1. Figure S2. Figure S3. Figure S4. [file CAM4-12-10768-s001.docx]

**Supplementary Figure 1. Shc3 expression is associated with P-gp in breast cancer A** Analysis the positively correlation between Shc3 and P-gp/ABCB1 on 1221 patients with breast cancer in R2 database (Mixed Breast (2022-v32) - tcga - 1221 - tpm - gencode36), Pearson's correlation coefficient 0.2, *P* < 0.0001. **B** Kaplan-Meier correlation analysis was conducted on 136 patients with breast cancer in R2 database to assess the relationship between Shc3 expression levels and overall survival (*P* = 0.0035).


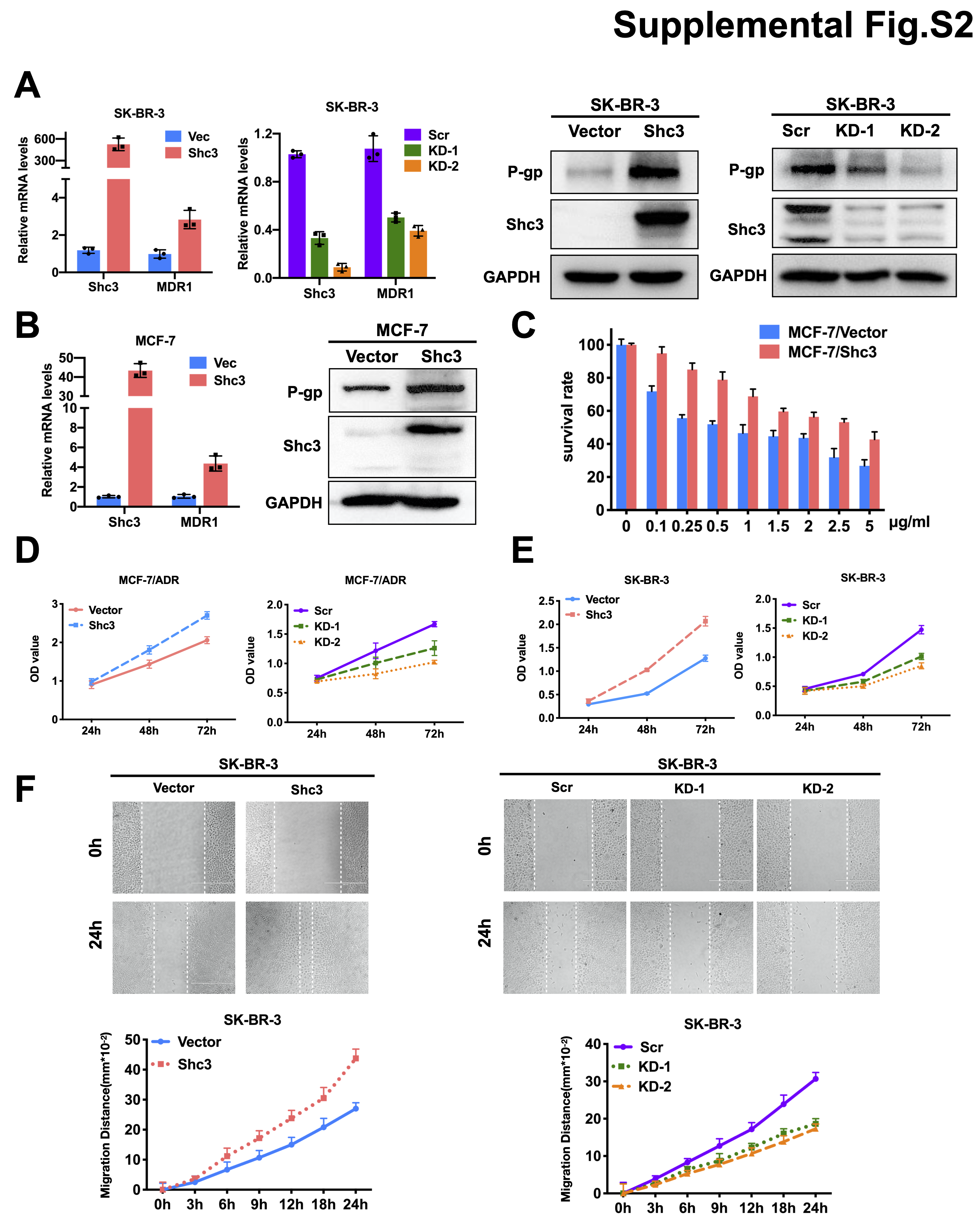


**Supplementary Figure 2. Shc3 induces chemoresistance and aggressive behavior in breast cancer cells. A** The mRNA and protein expression levels of Shc3 and MDR1 in SK-BR-3 stable overexpression and control cells were examined using qRT-PCR and western blot analysis. **B** The mRNA and protein expression levels of Shc3 and MDR1 in MCF-7 stable overexpression and control cells were examined using qRT-PCR and western blot analysis. **C** The cytotoxicity of doxorubicin in MCF-7 cells with stable Shc3 overexpression was evaluated by CCK8 assay and compared with that in the corresponding control cells. The IC_50_ values were respectively 0.6617 μg/mL in MCF-7//Vec cells and 2.975 μg/mL in MCF-7/Shc3 cells at 48h. **D** MCF-7/ADR Shc3-overexpressing and knockdown cells were detected by CCK8 assay. **E** Shc3-overexpressing and knockdown SK-BR-3 cells were detected by CCK8 assay. **F** The wound healing of SK-BR-3 Shc3-overexpressing and Shc3-KD cells was qualitatively recorded (images was shown at 0 hours and 24 hours, magnification: 100×).

**
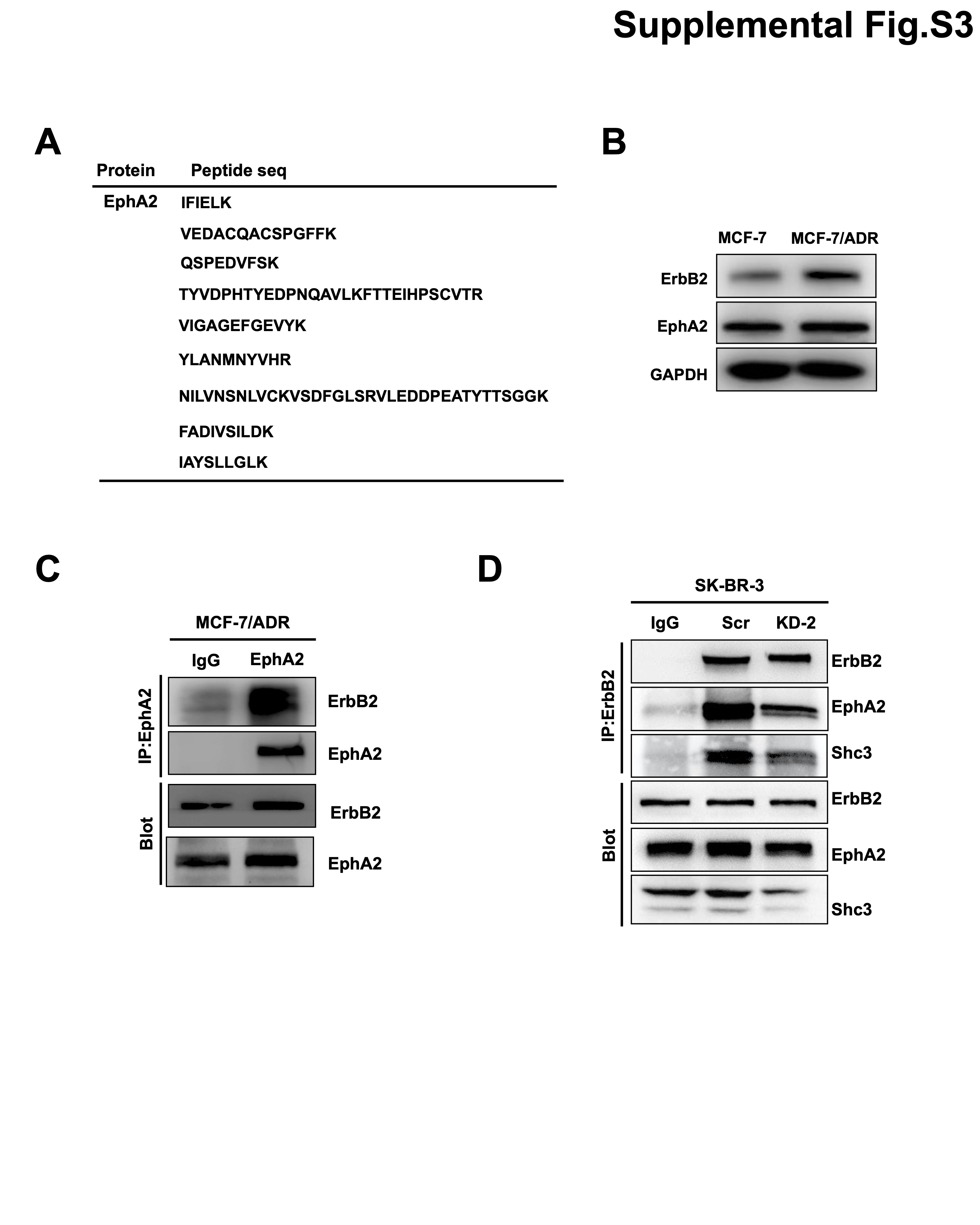
**

**Supplementary Figure 3. Shc3 interacts with EphA2 and ErbB2.** **A** Peptide sequences of EphA2 interacting with Shc3 in MCF-7/ADR cells identified in MS. **B** Using western blot assay to exmaine the expression of ErbB2, EphA2, GAPDH in MCF-7 and MCF-7/ADR cells. **C** Co-IP assay shown that EphA2 associates with ErbB2 in MCF-7/ADR cells. **D** In SK-BR-3 cells, Shc3 down-regulation impaired the interaction between EphA2 and ErbB2. Lysates of Shc3 knockdown cells and vector control cells were immunoprecipitated with anti-ErbB2 antibodies and control IgG antibodies.

**Supplementary Figure 4. Shc3 promotes nuclear localization of ErbB2. A** Western blot was performed to measure the relative total, cytoplasmic and nuclear expression levels of P-gp and ErbB2 in SK-BR-3/Vector and SK-BR-3/Shc3 cells. GAPDH was used as the cytoplasmic control; Histone-3 was used as the nuclear control.
