# Supplementary material for: Shc3 facilitates breast cancer drug resistance by interacting with ErbB2 to initiate ErbB2/COX2/MDR1 axis
Source: Cancer Med. 2023 Mar 7;12(9):10768–80. doi: 10.1002/cam4.5768 (PMC10225176; doi:10.1002/cam4.5768)
Supplement: Supplementary file 2 — Table S1. Table S2. Table S3. Table S4. [file CAM4-12-10768-s003.docx]

**Supplemental tables**

**Table S1. Sequences of the genes coding shRNA for Shc3 knockdown experiments.**

| Name | Target sequence |
| --- | --- |
| Shc3-KD1 | CTCCGGTTTAAGCAATATTTA |
| Shc3-KD2 | GCTCCGGTTTAAGCAATATTT |
| shControl | TCCTAAGGTTAAGTCGCCCTC |

**Table S2. Sequences of the genes coding siRNAs for ErbB2 knockdown experiments.**

| Name | Target sequence |
| --- | --- |
| siErbB2 | GCTCTTTGAGGACAACTAT |

**Table S3. Sequences of the genes coding siRNAs for COX2 knockdown experiments.**

| Name | Target sequence |
| --- | --- |
| siCOX2-1 | TGAAAGGACTTATGGGTAA |
| siCOX2-2 | GGACTTATGGGTAATGTTA |

**Table S4. Sequences of the DNA primers for qRT-PCR.**

| Name | Sequence (5’-3’) |
| --- | --- |
| Shc1 | Forward: GGGTGTGGTTCGGACTAAGG  Reverse: AGGGCAGATCACAGTTTCCG |
| Shc2 | Forward: CAGCTTCATCCACAAACCCG  Reverse: GCATAGAGCGGAGAACCTCG |
| Shc3 | Forward: AAGCCTTTGAGCTCCGGTTT  Reverse: GGAGGAGGCATCTTGCTTG |
| Shc4 | Forward: GCTGTGGTCTCACTCTAGCTG  Reverse: CATGTGTACTGCTGACACCATTTA |
| GAPDH | Forward: TGCACCACCAACTGCTTAGC  Reverse: GGCATGGACTGTGGTCATGAG |
| MDR1 | Forward: CCCATCATTGCAATAGCAGG  Reverse: GTTCAAACTTCTGCTCCTGA |
| COX2 | Forward: TTCAAATGAGATTGTGGAAAAAT  Reverse: AGATCATCTCTGCCTGAGTATCTT |
| ChIP-qPCR of COX2 | Forward: CTTCAAAATAAGCTTGAATTCAGGATTGTAATG  Reverse: CTTTTTGATAATTTAATAATTTCAATCTTCTGTTTC |
