# Supplementary material for: Shc3 facilitates breast cancer drug resistance by interacting with ErbB2 to initiate ErbB2/COX2/MDR1 axis
Source: Cancer Med. 2023 Mar 7;12(9):10768–80. doi: 10.1002/cam4.5768 (PMC10225176; doi:10.1002/cam4.5768)
Supplement: Supplementary file 3 — Data S1. [file CAM4-12-10768-s002.docx]

**1. Materials and methods**

**1.1 RNA sequencing (RNA-seq) data analysis**

RNA integrity was assessed using an RNA Nano 6000 Assay Kit for the Bioanalyzer 2100 system (Agilent Technologies, CA, USA). Total RNA was used as input for the RNA sample preparation. Transcript abundance is quantified with Salmon from fastq files [1]. Differential expression analysis between the two groups was performed using the DESeq2 [2]. Genes with an adjusted *P*-value <0.05 determined by DESeq2 were defined as differentially expressed.

**1.2 Antibodies**

The antibodies used for western blotting were as follows: anti-Shc3 and anti-GAPDH (Santa Cruz Biotechnology, CA); anti-P-gp, anti-ErbB2, and anti-H-3 (Abcam, UK); anti-EphA2, anti-p-c-Raf, anti-c-Raf, anti-p-MEK, anti-MEK, anti-p-ERK, anti-ERK, anti-p-Akt, anti-Akt, and anti-COX-2 (Cell Signaling Technology, USA).

Antibodies specific for Shc3 (1:100, Santa Cruz Biotechnology, CA), P-gp (1:250, Abcam, UK) and ErbB2 (1:500, Abcam, UK) were used for routine IHC staining with the Dako EnVision two-step method in accordance with the manufacturer’s instructions.

**1.3 Immunofluorescence assay**

MCF/ADR-Vector and MCF/ADR-Shc3 cells were seeded in 12-well plates containing sterile coverslips and cultured for 24 hours. Cells were fixed with 4% paraformaldehyde for 10 min at room temperature and permeabilized with 0.1% Triton X-100 for 10 min. Blocking of nonspecific binding sites was performed by incubation in 5% BSA in PBS for 1 hour. Then, the cells were incubated with the anti-ErbB2 antibody (Abcam, UK) for 1 hour and then with 1 μg/ml 4′,6-diamidino-2-phenylindole (DAPI) (1:1000 dilution) for 10 min at room temperature (RT). Morphological features were quantified using confocal laser scanning microscopy (Olympus FV1000).

**References**

1. Patro R, Duggal G, Love MI, Irizarry RA, Kingsford C: Salmon provides fast and bias-aware quantification of transcript expression. *Nat Methods* 2017, 14(4):417-419.

2. Love MI, Huber W, Anders S: Moderated estimation of fold change and dispersion for RNA-seq data with DESeq2. *Genome Biol* 2014, 15(12):550.
